# Drug-Induced Regulation of Target Expression

**DOI:** 10.1371/journal.pcbi.1000925

**Published:** 2010-09-09

**Authors:** Murat Iskar, Monica Campillos, Michael Kuhn, Lars Juhl Jensen, Vera van Noort, Peer Bork

**Affiliations:** 1 Structural and Computational Biology Unit, European Molecular Biology Laboratory, Heidelberg, Germany; 2 Novo Nordisk Foundation Center for Protein Research, Faculty of Health Sciences, University of Copenhagen, Copenhagen, Denmark; 3 Max-Delbrück-Centre for Molecular Medicine, Berlin, Germany; Sony Computer Science Laboratories, Japan

## Abstract

Drug perturbations of human cells lead to complex responses upon target binding. One of the known mechanisms is a (positive or negative) feedback loop that adjusts the expression level of the respective target protein. To quantify this mechanism systems-wide in an unbiased way, drug-induced differential expression of drug target mRNA was examined in three cell lines using the Connectivity Map. To overcome various biases in this valuable resource, we have developed a computational normalization and scoring procedure that is applicable to gene expression recording upon heterogeneous drug treatments. In 1290 drug-target relations, corresponding to 466 drugs acting on 167 drug targets studied, 8% of the targets are subject to regulation at the mRNA level. We confirmed systematically that in particular G-protein coupled receptors, when serving as known targets, are regulated upon drug treatment. We further newly identified drug-induced differential regulation of Lanosterol 14-alpha demethylase, Endoplasmin, DNA topoisomerase 2-alpha and Calmodulin 1. The feedback regulation in these and other targets is likely to be relevant for the success or failure of the molecular intervention.

## Author Summary

Many drug targets thought to be suitable for therapeutic purposes are subjected to positive or negative feedback loops upon chemical perturbations which might even account for the development of drug tolerance. In this study, we carried out the first systematic analysis of drug-induced differential expression of drug targets using the Connectivity Map, a resource that contains the genome-wide expression profiles of 1309 bioactive small molecules performed on four cultured human cells. The main obstacle in analyzing such a large set of profiles is the non-biological experimental variation across batches. We overcame this by developing a pipeline for strict filtering and state-of-the-art normalization and were able to utilize the Connectivity Map for assessing the drug-induced differential regulation of drug targets. Using the normalized data, we found that at least 8% of the drug-induced drug targets studied are differentially regulated in three cell lines; some of these confirm previous observations in other cell lines. Our work not only quantifies the amount of target expression feedback loops in three human cell lines, but also identifies so far unknown drug-induced target expression changes; some of them can be linked to the development of drug tolerance in patients.

## Introduction

For the future development of new drugs, the understanding of their mechanisms of action is vital. To tackle this in a large-scale, systemic way, the Connectivity Map (CMap) consortium studied the effects of 1309 bioactive small molecules including more than 800 marketed drugs on genome-wide gene expression in four cultured human cells, [1] (http://www.broadinstitute.org/cmap/). Although drugs can perturb biological systems by interacting with different types of biomolecules [2], analysis of successful drugs has shown that generally they bind and alter the activity of proteins (so called drug targets). The monitoring of genome-wide gene expression is likely to reveal insights into the action of drugs and the prediction of additional drug targets [1], [3].

One important aspect of a good target is its reliability and vulnerability over long periods. Biological systems are robust in a way that they restore the perturbations caused by drug treatments. Many drug targets thought to be suitable for therapeutic purposes turn out to be less effective than expected or account for adverse side effects [4]. Overcoming biological robustness, maintained through positive or negative feedback loops of the drug target proteins, might be a key factor for success of the intended therapeutic usage of drugs [4], [5]. The genome-wide transcriptional profiling using microarrays [1] should enable us to specifically monitor the expression changes of drug targets induced by their inhibitors or activators. The essential data required for this data integration are provided by i) STITCH: a drug-target relations resource [6] and ii) the Connectivity Map (CMap) which contains genome-wide expression profiles of cells treated with small-molecules [1].

STITCH [6] is a repository merging multiple sources of protein-chemical interactions providing ‘actions’ (inhibition/activation) for 81% of the human chemical-protein interactions. Of those, 1290 drug-target interactions are present in the CMap comprising the actions of 466 drugs on 167 drug targets.

CMap is a searchable database of gene expression profiles [1] that builds on the success of gene expression profiles from diverse chemical compounds in predicting the toxicity and/or mechanism of action of a drug [7], [8]. CMap data have been already used to create a human drug-drug and disease-drug network [9], [10]. The similarity of gene expression profiles recorded for unrelated stimuli in cells grown at the same time (also called batch effect) is a phenomenon known for microarray studies that needs to be overcome [11]. In order to remedy the batch effect problem in CMap and to make CMap amendable to various large scale studies, Iorio *et al.* proposed to construct a ‘Prototype List’ of the drug by merging its experiments from cell lines, batches, concentrations and microarray platforms [9]. As the signal to noise ratio can still be further improved, we implement here a novel protocol with filtering and normalization steps in order to utilize CMap for the elucidation of drug-induced feedback mechanisms.

## Results/Discussion

### Data filtering and expression profile scoring

**Figure 1:**
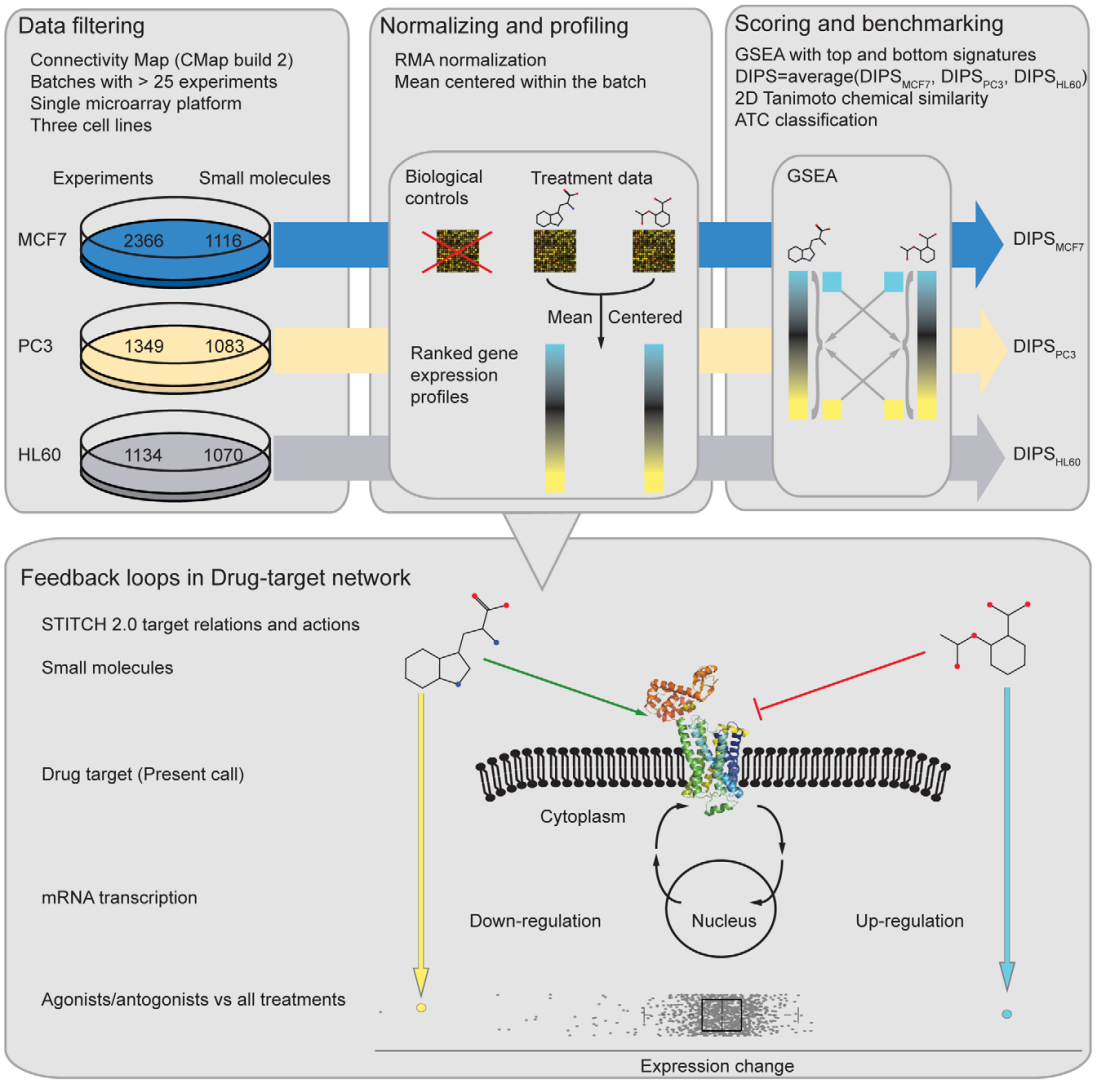
Workflow for the pipeline used to normalize and analyze Connectivity Map microarray experiments. The reliability of drug-induced gene expression profile similarity scores (DIPS scores) were evaluated using independent drug features as benchmark. Using the processed data, differential regulation of drug-induced drug targets was investigated.

We obtained reliable expression differences of drug targets by filtering and normalizing the gene expression profiles (Figure 1). In CMap, microarray experiments were collected from four cell lines treated with 1309 small molecules at different ranges of concentrations and only partially with replicates. We performed several filtering and normalization steps leaving a total of 1144 perturbations for further processing (Figure 1). After pre-processing, each probe in the drug-induced gene expression profiles was mean centered using the average of all drug perturbation experiments in the corresponding batch rather than using its biological controls. We calculated pair-wise drug-induced gene expression profile similarity (DIPS) scores using Gene set enrichment analysis (GSEA, [12] with a similar methodology as described in Iorio *et al.* [9], see Materials and Methods). In total, 4,349,432 DIPS scores were calculated between all drug pairs in three cell lines, that we used to compare gene-expression profiles of pairs of drugs.

### Background estimates and data normalization

**Figure 2:**
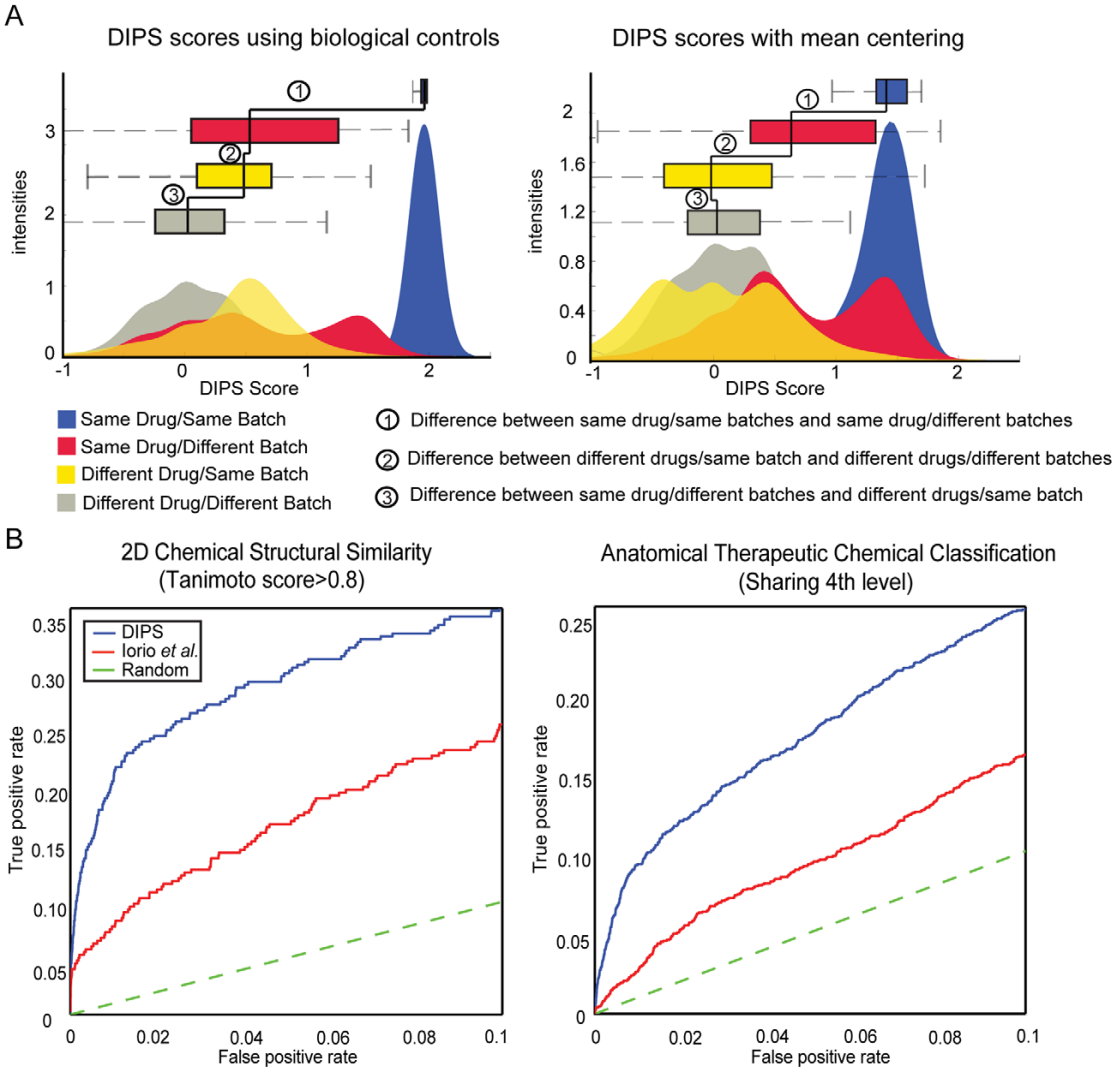
Analysis of systematic biases and benchmarking with independent features of chemicals. (**A**) Distributions of the DIPS scores for the pair-wise comparisons of gene expression profiles constructed using biological controls and mean centering as background across four drug/batch categories: i) both profiles are from the same drug and the same batch (Blue), ii) the same drug from different batches (Red), iii) different drugs from the same batch (Yellow) and iv) different drugs from different batches (Grey) (**B**) ROC curves are used to assess the performance of the DIPS score (blue line) and provide a comparison with the method described in Iorio *et al.* (red line) [9]. Area under the curve values for each ROC curve: Chemical structural similarity: AUC (DIPS = 0.028 for FPR<0.1) and (AUC Iorio *et al.* = 0.016 for FPR<0.1). For 4^th^ level ATC sharing, the AUC (DIPS = 0.016 for FPR<0.1) and (AUC Iorio *et al.* = 0.009 for FPR<0.1)(Refer to Figure S2 for the complete ROC plots.).

To reveal systematic biases, the DIPS scores of drug-induced gene expression profiles using biological controls were classified into four drug and batch categories (Figure 2A). The DIPS scores between different drugs in the same batch are significantly higher than between different drugs from different batches, implying a considerable batch effect as has already been hinted at in the original CMap publication [1] (Figure 2A, Label 3). Still, characteristic drug features are reflected in the gene expression profiles, i.e. the DIPS scores between the same drugs (Figure 2A, Blue and Red) are significantly higher than between different drugs from different batches (Figure 2A, Grey) (t-test p-values<2.2×10^−16^).

We utilized the large number of treatments to infer the background gene expression (by mean-centering) instead of the few biological controls provided by CMap, in order to eliminate the batch effect. In this way, also common (e.g. stress) responses will be down-weighted to reveal the characteristic expression response of each chemical perturbation.

After this normalization the batch effect was largely eliminated and the data reflect the characteristic features of drug perturbations better. The DIPS scores between different drugs from the same batch are no longer higher than between different drugs from different batches (Figure 2A, Label 3). Additionally, the DIPS scores between the same drugs from different batches (Figure 2A, Red) are higher than the between different drugs from the same batch (Figure 2A, Yellow), revealing the concordance of drug-induced gene expression profiles across batches (t-test p-values<2.2×10^−16^). Same conclusions were also derived from the distributions of Pearson correlations for drug-induced gene expression profiles across four drug/batch categories (Figure S1).

### Assessment of the drug-induced gene expression profile similarity score

We prove the integrity and reliability of the homogeneous gene expression profiles constructed with mean centering, by employing benchmark sets representing different features of drugs such as chemical structure similarity and shared Anatomical Therapeutic Chemical (ATC) classification of the World Health Organization (WHO) [13]. Chemical structural similarity is an indicator of shared drug targets and mechanism of action [14]–[16]. It is reported that high chemical similarity (i.e. with Tanimoto 2D coefficients >0.85) tends to result in similar biological responses [17]. The ATC classification is based on both the therapeutic and chemical properties of the drug also referred to as the drug mode of action. Thus, we expect that pairs of drugs with high structural similarity or shared ATC classification result in similar gene expression profiles.

Benchmarking shows that the DIPS scores calculated using the mean-centered procedure are clearly superior to the method proposed by Iorio *et al.* (Figure 2B). The area under the Receiver operator characteristic (ROC) curve (AUC) for the combined DIPS scores for 989 drugs (average over three cell lines), are higher both when using chemical similarity (Tanimoto 2D coefficient >0.8) and the 4^th^ level of the available ATC code shared between drug pairs (Figure S2). This confirms that the mean-centered data reflect the specific response after drug perturbation better than the treatment-control comparisons that were used previously [9].

Finally, the drug induced gene expression profiles were found to be concordant across cell lines (Figure S3). Although only cancer cell lines were used in CMap, the procedure proposed here should be applicable to drug perturbation profiles across multiple tissues and even organ systems.

### Differential expression of drug-induced drug targets

**Figure 3:**
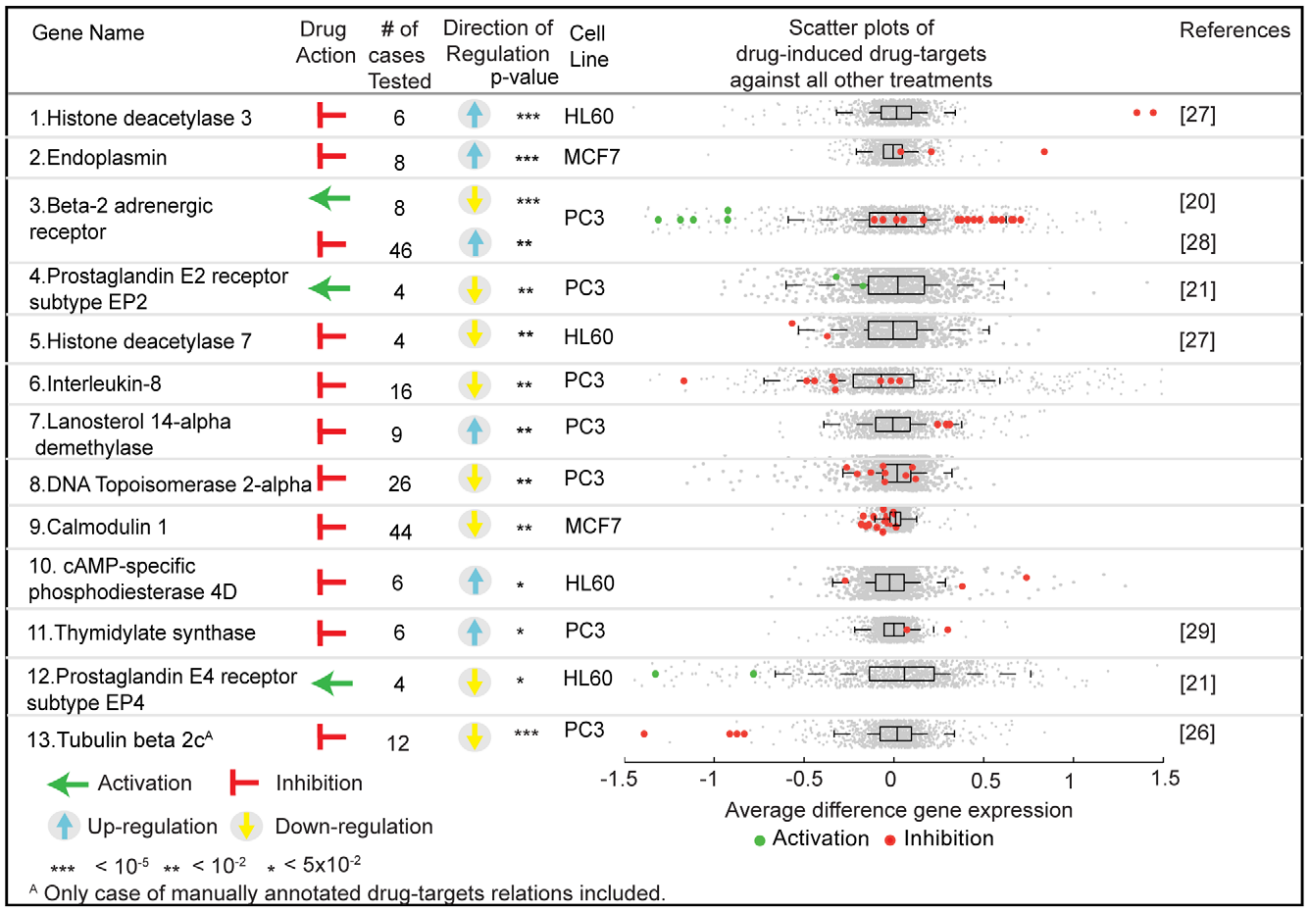
Drug-induced differentially regulated drug targets. Anova is used to assess the significance of the differential expression of drug-induced drug targets against the mRNA changes of the same gene in the population of heterogeneous drug treatments from CMap. The genes are mainly ordered based on their q-values as provided in Table S1. In the scatter plots, inhibitors/activators are labeled in red/green respectively and grey represents all other treatments present in CMap.

Integrating 4849 CMap arrays with 40,656 drug target relations from STITCH resulted in a set of 1,290 drug-target relations for which a genome-wide cellular response is available. We found that thirteen out of 167 distinct drug targets in this set (8%; 86 drug target relations) are subjected to significant differential expression upon drug treatment (Figure 3) by comparing the drug-induced expression changes of the drug target against all other treatments present in CMap (see methods). We found supporting evidence in the literature for seven out of thirteen (q-value <0.05) significant differential regulations of drug targets shown in Figure 3, confirming the rationale and predictive power of our systematic approach. For the remaining six targets we can predict a hitherto unknown drug-induced differential regulation.

The identified, differentially regulated drug targets are enriched in G-protein coupled receptors (GPCRs) (Figure S4), in agreement with previous reports that members of the GPCR family are generally regulated by several mechanisms including receptor desensitization, endocytosis at the protein level and regulation of the cellular receptor content [18], [19]. In the three cancer cell lines used, we observe agonist-induced down-regulation of GPCR mRNAs for beta-2 adrenergic receptor (ADRB2), prostaglandin E2 receptor subtype EP2 and prostaglandin E4 receptor subtype EP4 (Figure 3, Genes 3,4,12), which were previously reported in DDT1 MF-2 smooth muscle cells (ADRB2) and 293-EBNA human embryonic kidney cells (prostaglandin E2/E4 receptor subtypes EP2/EP4) [20], [21]. This indicates that drugs can induce similar feedback loops in a wide variety of cell types. However, we cannot rule out that cross-regulation among signaling pathways may be responsible for the regulation of GPCR mRNAs as it has been described before [22]. For example, it has been shown that a beta adrenergic mRNA-binding protein, ELAV-like protein 1 (ELAVL1) can be induced by ADRB2 agonist or elevated levels of cyclic adenosine monophosphate (cAMP) [22], [23] and destabilizes ADRB2 mRNA. The ELAVL1 protein binds to GPCR mRNAs and recognizes a cognate sequence located at the 3′-UTR of ADRB2, proteinase-activated receptor and M2, M3 muscarinic acetylcholine receptor mRNAs [24], [25]. Therefore cAMP provides cross-talk among GPCR regulatory networks. However, it is shown that intracellular cAMP accumulation is not the only factor contributing to the reduction of ADRB2 mRNA levels [20]. Moreover, we find that GPCR-targeting drugs regulate the transcription of their specific targets (Figure S5). We conclude that in addition to the cross-regulation of G-protein signaling pathways drug target-specific feedback loops are also responsible for the regulation of drug target mRNAs.

In addition to cross-regulation of multiple drugs through the same signaling pathways, promiscuous drugs targeting multiple proteins may cause complex regulatory networks. In order to explore the cross-regulation of drug targets induced by a promiscuous drug, we searched and found that 259 out of 466 total drugs are multi-target drugs and 4 of these drugs act on multiple differentially regulated drug targets upon drug treatment. For example, Podophyllotoxin used in various chemotherapies is known to target both tubulin beta 2C and DNA topoisomerase 2-alpha. The tubulin beta 2C and DNA topoisomerase 2-alpha mRNAs are both down-regulated upon drug treatment in three cell lines (Figure 3, Genes 8,13). Tubulin beta 2C inhibitors induce microtubule depolymerization that leads to the specific down-regulation of tubulin beta 2C mRNAs preventing the translational synthesis and thus the further accumulation of abundant tubulin monomers [26]. Moreover, we found that DNA topoisomerase 2-alpha mRNAs are not down-regulated upon treatment of other tubulin inhibitors (Figure S5). Therefore, we conclude that feedback loops of tubulin beta 2C and DNA topoisomerase 2-alpha are not cross-regulated. Two other examples of multi-target drugs are vorinostat used for the treatment of cutaneous T cell lymphoma and trichostatin A that serves as an antifungal antibiotic. Vorinostat and trichostatin A are considered to be nonspecific histone deacetylase inhibitors. These drugs lead to the up-regulation of histone deacetylase 3 (HDAC3) and down-regulation of histone deacetylase 7 (HDAC7) (Figure 3, Genes 1,5). In this case it is unclear whether there is cross-regulation, although HDAC7 siRNA experiments failed to induce the up-regulation of HDAC3 mRNAs [27], a result that is disfavoring the cross-regulation.

While the above cases only confirm literature reports in other cell lines or tissues, we also identified new cases of drug-induced expression regulation of drug targets. The significant novel findings are the inhibitor-induced down-regulation of calmodulin 1, DNA topoisomerase 2-alpha and up-regulation of endoplasmin, lanosterol 14-alpha demethylase and cAMP-specific phosphodiesterase 4D (Figure 3, Genes 9,8,2,7,10). Lanosterol 14-alpha demethylase is actually an off-target of antifungal drugs that bind the mammalian version with lower affinity than the fungal lanosterol 14-alpha demethylase. Probably, the up-regulation of the mammalian lanosterol 14-alpha demethylase compensates for the undesired inhibition and modulates the adverse effects. On the contrary, we observed a feedback loop that accelerates the down regulation of calmodulin 1 mRNA induced by calmodulin inhibitors. Calmodulin targeting drugs can provide a rapid and effective therapeutic effect, while at the same time small variations of drug concentrations can increase adverse effects. Therefore, it would be interesting to study further the functional effects upon target inhibition of lanosterol 14-alpha demethylase, endoplasmin and calmodulin 1 to elucidate the roles of feedback loops in drug mode of action and adverse effects.

Drug-induced target regulation might be implicated in tolerance development and thus identifying potential target regulation should be an integral part of drug discovery to prevent failures in later stages of clinical trials. For example, we have observed the inhibitor-induced up-regulation of ADRB2 and thymidylate synthetase (TYMS)(Figure 3, Genes 3,11) [28]. TYMS is an essential enzyme for DNA replication/repair and an important drug target in cancerous cells. Indeed, it has been shown that inhibitor-induced TYMS over-expression obstructs the clinical efficiency by inducing tumor drug resistance [29]. In addition to over-expression, down-regulation of drug targets upon agonist treatment may also cause treatment tolerance as observed for ADRB2 long-acting agonist treatment. ADRB2 is a therapeutic target activated to treat the symptoms of asthma. We observe the agonist-induced up-regulation of ADRB2 and already in 2005, the FDA warned patients that ADRB2 might be down-regulated (desensitization) and be unresponsive for asthma treatment due to long-acting agonist exposure [20], [30]. Thus, robustness in biological systems could prevent the applicability of the long-term treatments via positive/negative feedback loops of the drug target affecting the clinical efficiency of drugs in trial and on the market. Drug-induced regulation of drug targets can thus be linked to tolerance development, which restricts the efficiency of clinical treatments where the drug concentration is limited to avoid an excess of adverse drug reactions.

Taken together, we have identified drug-induced differential regulation of drug targets. Due to the limited signal to noise ratio in the data at hand, the identified 8% of all drug targets that show feedback loops has to be seen as a lower limit, i.e. target-regulation appears as a wide-spread biological phenomenon that has to be taken into account during drug development.

## Materials and Methods

### Data source

Connectivity Map (CMap, build 02) contains 6100 gene expression profiles of 4 cell lines treated with 1309 distinct small molecules. Data from Connectivity Map was downloaded from the CMAP main website (http://www.broadinstitute.org/cmap/). On this data set, filtering was performed in multiple steps as follows: Treatment instances (i.e. one treatment versus vehicle control pair) from three cell lines (HL60, Human promyelocytic leukemia cell line; MCF7, Human breast adenocarcinoma cell line; PC3, Human prostate cancer cell line) were taken into consideration. Only the treatment instances from the production batches containing over twenty-five treatments in HT_HG-U133A platform were selected (with the exception of HL60 cell line where HG-U133A microarray platform was also included.) Lastly, for each cell line, the highest concentration of treatments was selected discarding lower concentration treatments. Figure 1 shows the number of treatment instances used in this study before and after filtering. In total, we analyze here a total of 4849 treatment instances in three cell lines corresponding to 1144 small molecules, in which 989 of them are tested in each cell line.

### Data pre-processing

Treatment arrays were grouped based on the cell line. For the HL60 cell line, treatments from different microarray platforms were further classified in separate groups. Each group was pre-processed separately using RMA [31]. Vehicle controls from CMap were discarded and for each batch individual probes of each treatment were mean centered to calculate the average difference values within the batch. To construct a unique gene expression profile of a small molecule for each cell line, replicate treatments were merged into one averaging their probe sets values. For the HL60 cell line, profiles from multiple microarray platforms were not merged because there was not any experiment with the same drug treatment from different microarray platforms.

The probe sets for the small-molecule gene expression profile were ranked based on both their detection call and their average log-signal difference value for the probe set [32]. Detection calls were assigned on the probe sets for individual experiments. A probe set was labeled to be ‘Present in a cell line’ if the detection call algorithm had assigned ‘present’ for that probe set in at least half of the drug treatment experiments in that corresponding cell line. Ranking was performed in two steps. First, for probe sets assigned to be ‘Absent’ in tested cell line, the average difference was set to 0. Next, all probe sets were ranked in the descending order of their average difference. Last, to get the final ranked gene expression profile, the probe sets, which were set to 0, were sub-sorted based on their initial average difference.

### Pairwise similarity score of drugs

Pairwise similarity scores between small-molecule gene expression profiles were calculated using a similar method presented in Iorio *et al.* [9]. An optimal signature was created for each gene expression profile of the drug. This optimal signature consists of the top 250 and bottom 250 ranked probe sets in the gene expression profile. These probe sets are the characteristic cellular response of the drug treatment that might be specific to cell line. To get the similarity score between drug X and Y: The down-regulated and up-regulated features of the optimal signature from drug X were searched within the weighted gene expression profile of drug Y. In same respect, top and down regulated signature genes of drug Y were also searched within the weighted gene expression profile of Drug X. To quantify a similarity score, gene set enrichment analysis (GSEA) based on Kolmogorov-Smirnov statistics were used [12]. All results obtained through GSEA were averaged to obtain the final score of gene expression profile comparison for a drug pair in a specific cell line.

As we might have up to three expression profiles of the small-molecule treatment from three cell lines, it is possible to compare gene expression profiles of the drug pairs within and between cell lines. In this study, drug-induced gene expression profile similarity scores were only calculated within cell lines. To increase reliability of DIPS score, a combined similarity score was calculated as the average of the similarity scores for the drug pair from multiple cell lines.

### Drug target expression changes

A drug can act on the protein if the protein is physically present in the cell. A drug target was considered to be ‘expressed’ and present if the detection call algorithm [32] reports it as such for one-tenth of the treatment experiments in the cell line. Drug targets that were not expressed and labeled “absent” were excluded. Drug target information was gathered from STITCH 2.0 including actions of the interaction. These interactions were labeled as ‘activation’ or ‘inhibition’ (including ‘binding’). To minimize indirect associations, only the drug-target relations from experimental and curated database annotations over 0.7 threshold were taken into consideration. For the significant cases of drug-induced differential regulation of drug targets, ‘binding’ associations are manually corrected and the results are re-calculated.

Drug-induced gene-expression of a drug target used in this context indicates the expression change of the target mRNA upon drug treatment acting on the corresponding target. Significance of the expression changes of the drug target were evaluated by comparing the drug-induced expression changes of target mRNAs with the expression changes of the target upon all other chemical treatments present in CMap. ANOVA was used to assess the significance for the expressional change of individual drug targets from multiple cell lines. Table S1 provides the *t*-test results for the expression changes of drug targets for individual cell lines.

## Supporting Information

Figure S1 Histograms comparing the distributions of pearson correlations for drug induced gene expression profiles across four drug/batch categories using two different pre-processing pipelines. Main difference of these two pipelines is based on the selection of control as background; biological controls or mean centering. In the case of using biological controls, the distribution of pearson correlations between different drugs in the same batch are significantly higher than between different drugs from different batches, indicating the batch effect within the data.
(0.17 MB TIF)

Figure S2 Same as Figure 2B of the main paper, complete ROC plots for DIPS score benchmarking with independent features of chemicals. (A) The AUC of DIPS and Iorio et al. are 0.70 and 0.62 respectively with the binary classification of tanimoto score over 0.8. (B) Shared 4th level of Anatomical Therapeutic Chemical classification is used as binary classifier for the drugs with available ATC classification. AUC of DIPS and Iorio et al. are 0.63 and 0.55, respectively.
(0.40 MB TIF)

Figure S3 Distributions of the drug-induced gene expression profile similarity scores for drug-drug pairs across four drug/cell line categories. To gain a comprehensive picture of drug concordance across cell lines, we evaluated whether the similarity scores are driven by cell-line specific gene expression. For pairwise comparison of profiles from different cell lines, drug treatments from multiple cell lines were merged into one matrix where only the ‘Present’ probe sets for three cell lines were preserved in the matrix. The DIPS scores on the category of the same drug from different cell lines (D+/C−) are significantly higher than the scores within the category of different drugs from the same cell line (D−/C+) (t-test p-value <2.2×10−16) meaning that the drug response is generally concordant across cell lines.
(0.34 MB TIF)

Figure S4 Distribution of molecular functions of differentially regulated drug-targets (p-value <0.05) compared to molecular functions of all other targets. Drug target classification is based on the categorization described in ChEMBL with few changes. For instance, for a more detail categorization, the lyase, hydrolase, oxidoreductase and transferase activities were included. If a drug target is identified within multiple categories, the order of the categories introduced was used to choose the first category as the main category to annotate the drug target. (GO:0004930: G-protein coupled receptor activity, GO:0004879: Ligand-dependent nuclear receptor activity, GO:0005216: Ion channel activity, GO:0005215: Transporter activity, GO:0004112: Cyclic-nucleotide phosphodiesterase activity, GO:0005198: Structural molecule activity, GO:0003700: Transcription factor activity, GO:0016301: Kinase activity, GO:0008233: Peptidase activity, GO:0016829: Lyase activity)
(0.97 MB TIF)

Figure S5 Differentially regulated GPCRs and topoisomerase II of Figure 3 were tested by other GPCR antagonist/agonists and tubulin inhibitors, respectively. The GPCR antagonists/agonists were selected based on the ‘present’ call for their targets in the tested cell line (see Materials and Methods). Both drug-induced receptor-specific response and cross-regulation among signaling pathways may be responsible for the differential regulation of drug targets.
(0.42 MB TIF)

Table S1 Complete list of statistical tests for the differential regulations of drug-targets. The average difference values for the drug-induced drug-targets are tested against the mRNA changes of the same gene in the population of heterogeneous drug treatments from CMap. Anova is used to assess the significance of the differential expression of drug-induced drug targets in multiple and specific cell lines, respectively. This excel sheet contains the name of the drug-targets with its evaluation in multiple cell lines. Only the drug-targets whose detection call are ‘Present’ in more than one-tenth of the experiments in specified cell line, are pursued for the analysis of differential gene-expression of drug-targets. If the detection call does not specify the required criteria, the column of a drug-target for that cell line is left blank. The q-values reported here are calculated using the FDR correction algorithm.
(0.10 MB XLS)
