# Supplementary figures and images for: Drug-Induced Regulation of Target Expression
Source: PLoS Comput Biol. 2010 Sep 9;6(9):e1000925. doi: 10.1371/journal.pcbi.1000925 (PMC2936514; doi:10.1371/journal.pcbi.1000925)

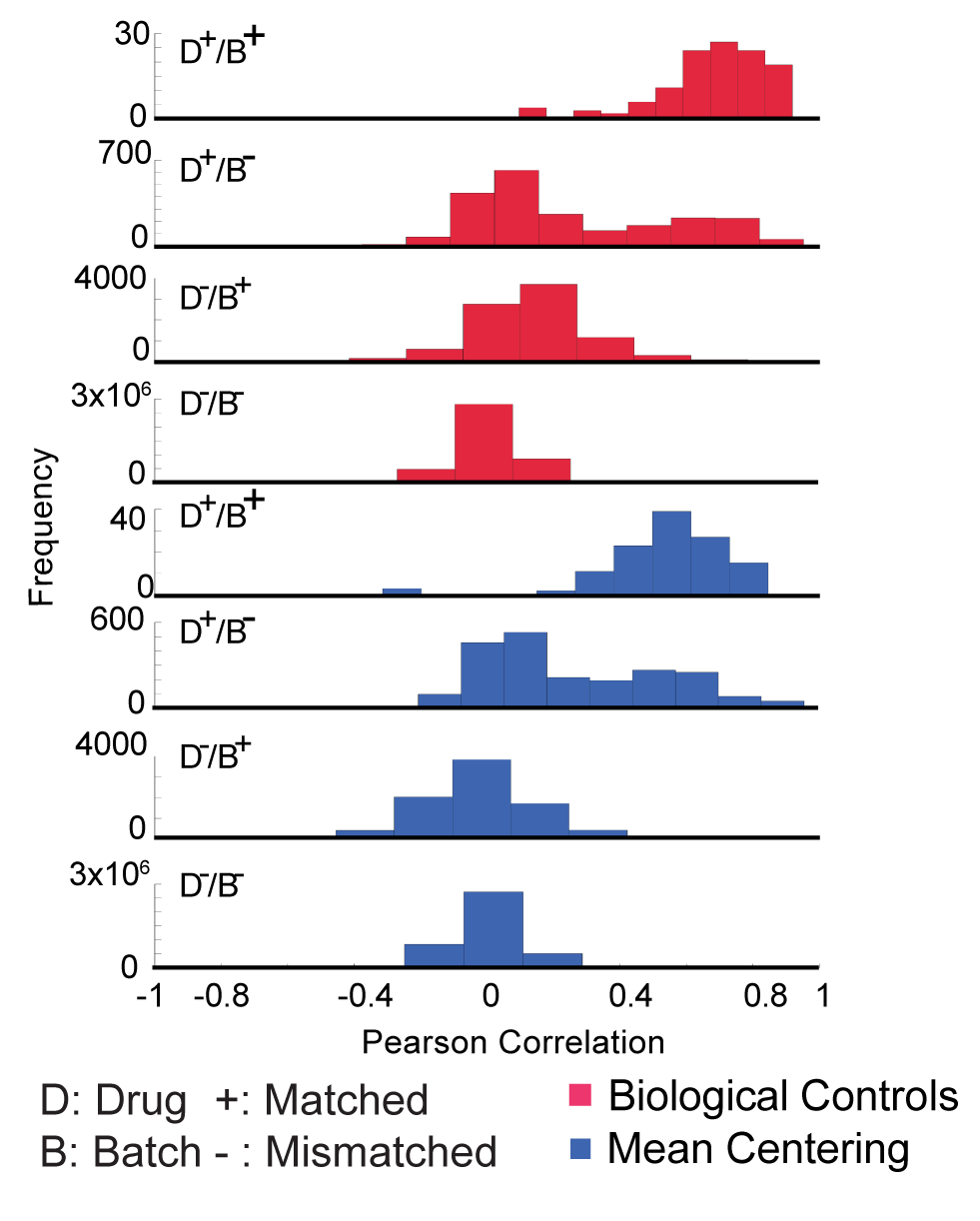

Supplement: Figure S1 — Histograms comparing the distributions of pearson correlations for drug induced gene expression profiles across four drug/batch categories using two different pre-processing pipelines. Main difference of these two pipelines is based on the selection of control as background; biological controls or mean centering. In the case of using biological controls, the distribution of pearson correlations between different drugs in the same batch are significantly higher than between different drugs from different batches, indicating the batch effect within the data. (0.17 MB TIF) [file pcbi.1000925.s001.tif]

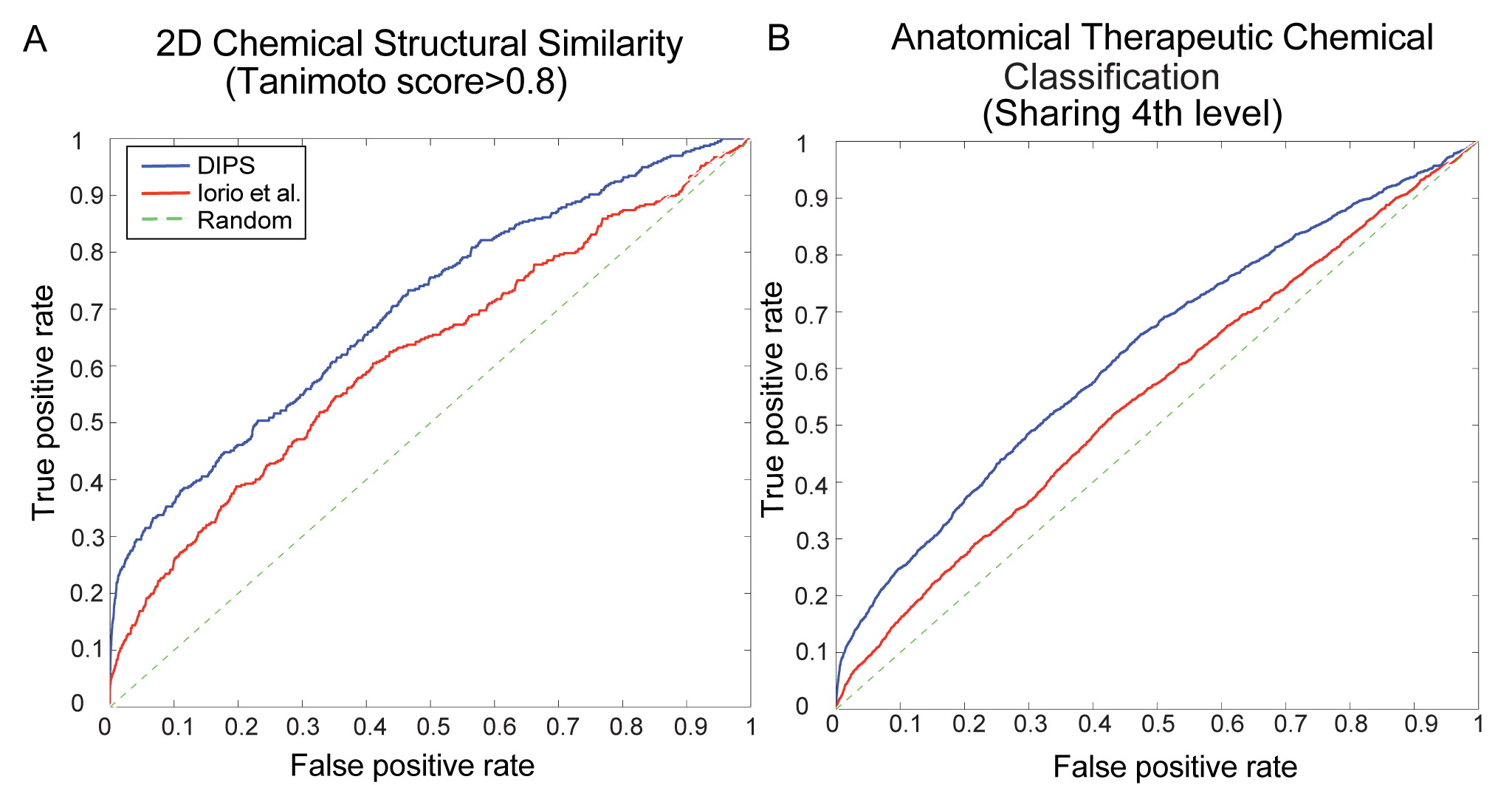

Supplement: Figure S2 — Same as Figure 2B of the main paper, complete ROC plots for DIPS score benchmarking with independent features of chemicals. (A) The AUC of DIPS and Iorio et al. are 0.70 and 0.62 respectively with the binary classification of tanimoto score over 0.8. (B) Shared 4th level of Anatomical Therapeutic Chemical classification is used as binary classifier for the drugs with available ATC classification. AUC of DIPS and Iorio et al. are 0.63 and 0.55, respectively. (0.40 MB TIF) [file pcbi.1000925.s002.tif]

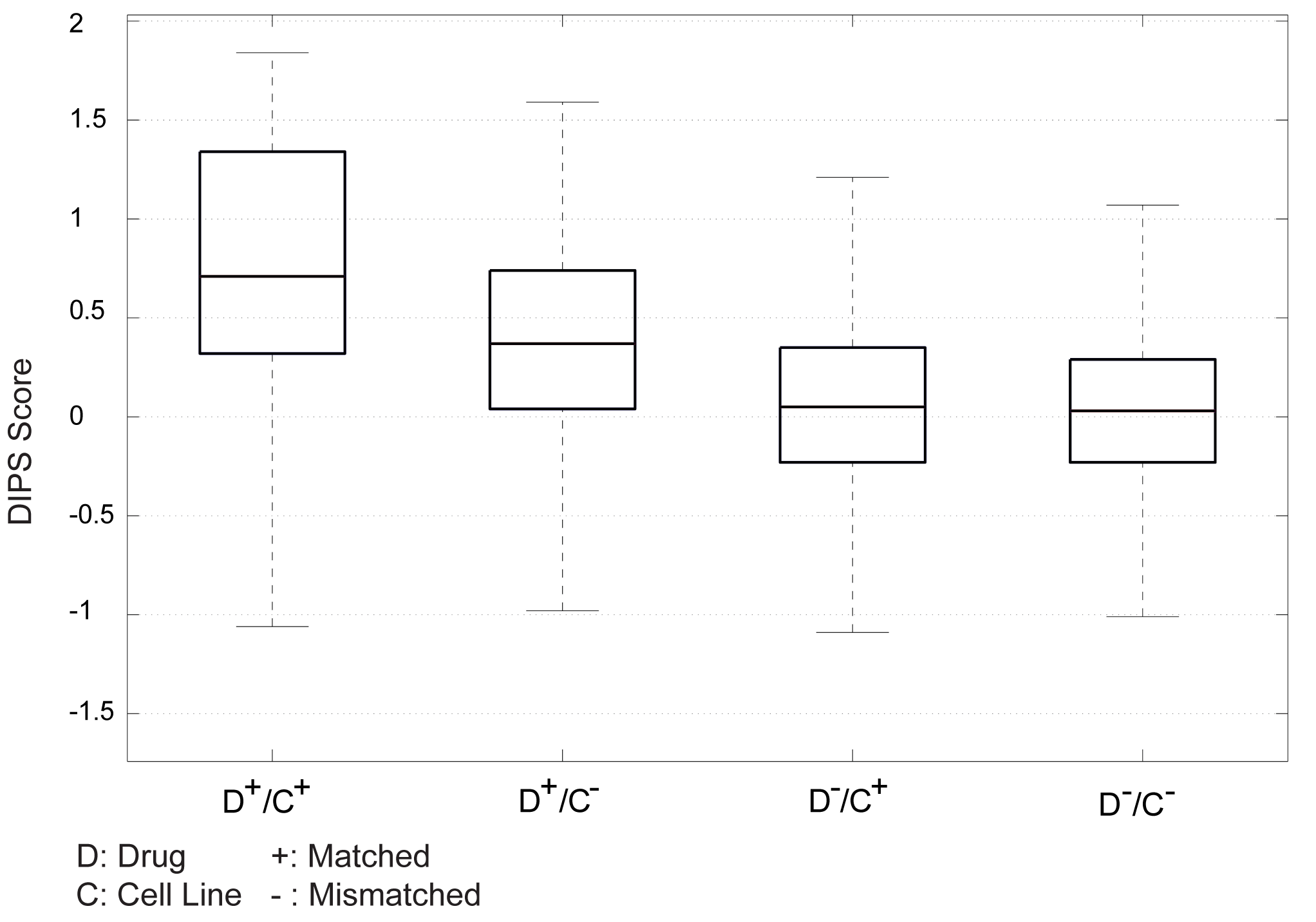

Supplement: Figure S3 — Distributions of the drug-induced gene expression profile similarity scores for drug-drug pairs across four drug/cell line categories. To gain a comprehensive picture of drug concordance across cell lines, we evaluated whether the similarity scores are driven by cell-line specific gene expression. For pairwise comparison of profiles from different cell lines, drug treatments from multiple cell lines were merged into one matrix where only the ‘Present’ probe sets for three cell lines were preserved in the matrix. The DIPS scores on the category of the same drug from different cell lines (D+/C−) are significantly higher than the scores within the category of different drugs from the same cell line (D−/C+) (t-test p-value <2.2×10^−16^) meaning that the drug response is generally concordant across cell lines. (0.34 MB TIF) [file pcbi.1000925.s003.tif]

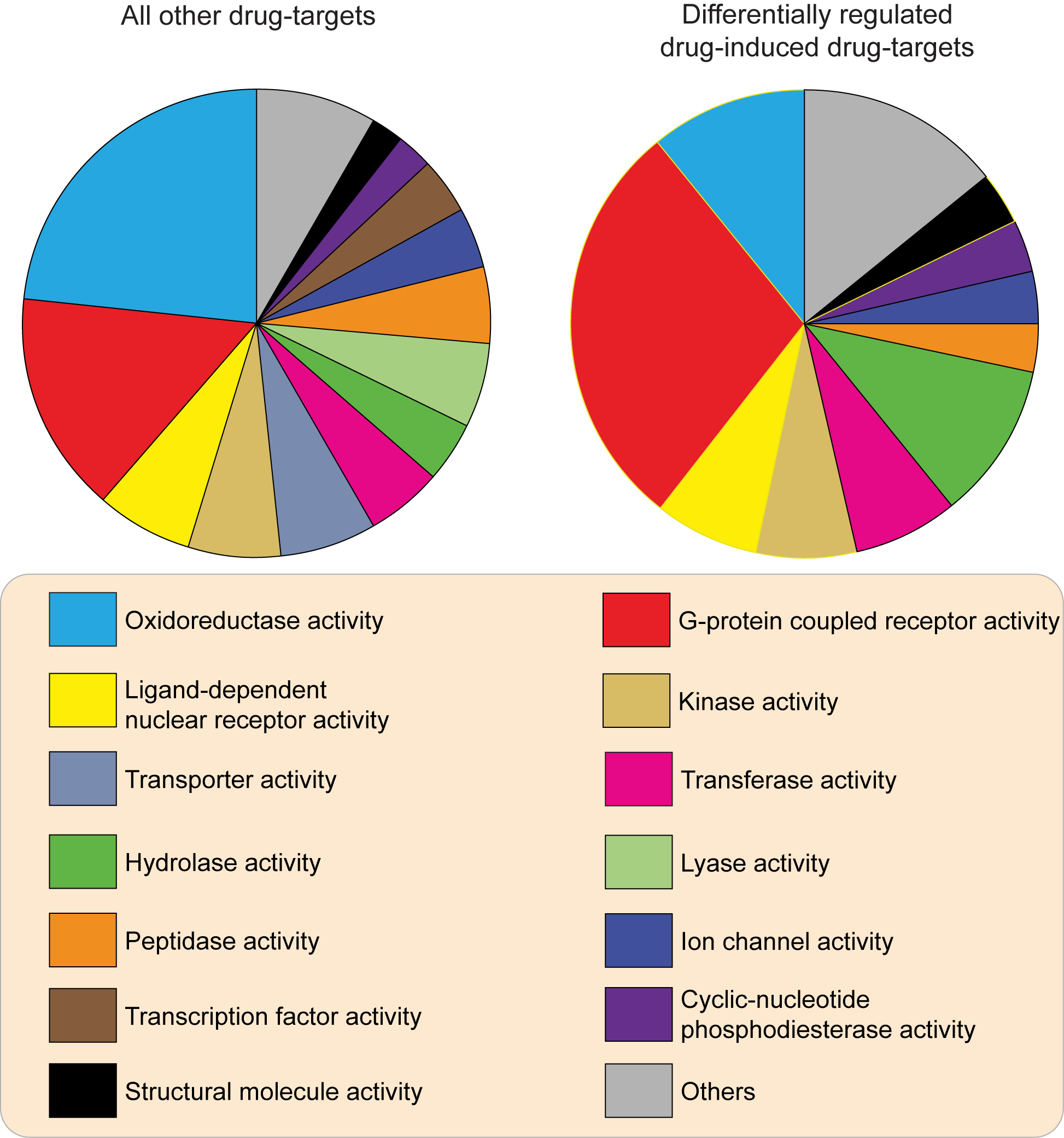

Supplement: Figure S4 — Distribution of molecular functions of differentially regulated drug-targets (p-value <0.05) compared to molecular functions of all other targets. Drug target classification is based on the categorization described in ChEMBL with few changes. For instance, for a more detail categorization, the lyase, hydrolase, oxidoreductase and transferase activities were included. If a drug target is identified within multiple categories, the order of the categories introduced was used to choose the first category as the main category to annotate the drug target. (GO:0004930: G-protein coupled receptor activity, GO:0004879: Ligand-dependent nuclear receptor activity, GO:0005216: Ion channel activity, GO:0005215: Transporter activity, GO:0004112: Cyclic-nucleotide phosphodiesterase activity, GO:0005198: Structural molecule activity, GO:0003700: Transcription factor activity, GO:0016301: Kinase activity, GO:0008233: Peptidase activity, GO:0016829: Lyase activity) (0.97 MB TIF) [file pcbi.1000925.s004.tif]

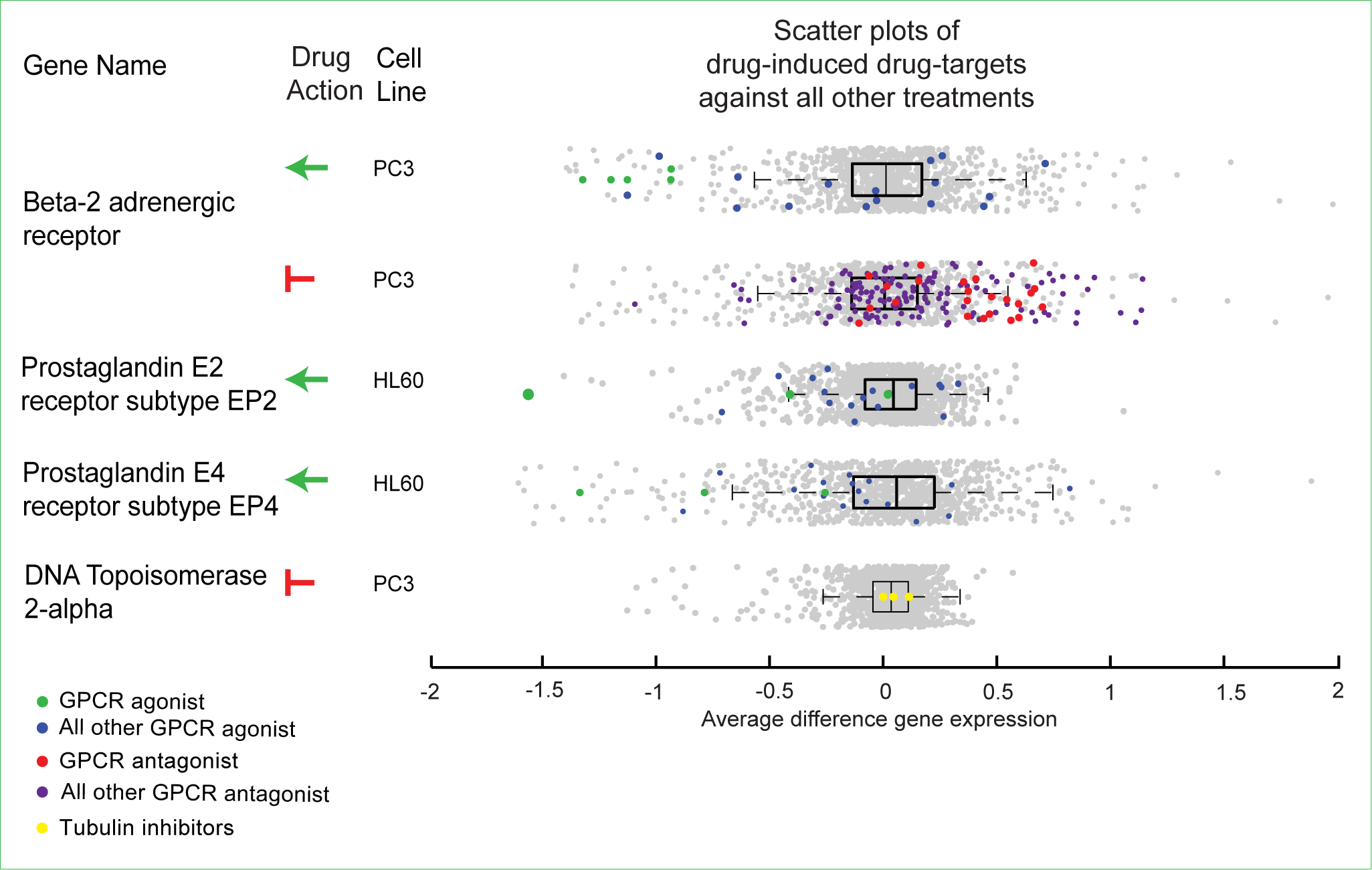

Supplement: Figure S5 — Differentially regulated GPCRs and topoisomerase II of Figure 3 were tested by other GPCR antagonist/agonists and tubulin inhibitors, respectively. The GPCR antagonists/agonists were selected based on the ‘present’ call for their targets in the tested cell line (see Materials and Methods). Both drug-induced receptor-specific response and cross-regulation among signaling pathways may be responsible for the differential regulation of drug targets. (0.42 MB TIF) [file pcbi.1000925.s005.tif]
